# Evaluation of Serum Cystatin C as a Marker of Early Renal Impairment in Patients with Liver Cirrhosis

**DOI:** 10.1155/2015/309042

**Published:** 2015-10-13

**Authors:** Mahmoud Omar, Wael Abdel-Razek, Gamal Abo-Raia, Medhat Assem, Gasser El-Azab

**Affiliations:** ^1^Department of Hepatology, National Liver Institute, Menoufia University, Shebeen El-Kom, Egypt; ^2^Department of Clinical Pathology, National Liver Institute, Menoufia University, Shebeen El-Kom, Egypt

## Abstract

*Background.* Serum cystatin C (CysC) was proposed as an effective reflection of the glomerular filtration rate (GFR). However, its role in patients with liver cirrhosis has not been extensively verified especially in the detection of early RI. *Patients and Methods.* Seventy consecutive potential candidates for living donor liver transplantation with serum creatinine (Cr) <1.5 mg/dL were included. CysC, Cr, and estimated GFR [creatinine clearance (CCr), Cockcroft-Gault formula (C-G), MDRD equations with 4 and 6 variables, CKD-EPI-Cr, CKD-EPI-CysC, and CKD-EPI-Cr-CysC] were all correlated to isotopic GFR. Early RI was defined as GFR of 60–89 mL/min/1.73 m^2^. *Results.* Patients were 25.7% and 74.3% Child-Pugh classes B and C, respectively. GFR was ≥90, 60–89, and 30–59 mL/min/1.73 m^2^ in 31.4%, 64.3%, and 4.3% of the patients, respectively. All markers and equations, except C-G, were significantly correlated to GFR with CKD-EPI-Cr-CysC formula having the highest correlation (*r* = 0.474) and the largest area under the ROC curve (0.808) for discriminating early RI. At a cutoff value of 1.2 mg/L, CysC was 89.6% sensitive and 63.6% specific in detecting early RI. *Conclusion.* In patients with liver cirrhosis, CysC and CysC-based equations showed the highest significant correlation to GFR and were measures that best discriminated early RI.

## 1. Introduction

Renal impairment is a common finding in patients with chronic liver disease; it has a huge impact on the patients' survival [1]. Moreover, the severity of renal dysfunction increases with the advancement of liver cirrhosis and portal hypertension [2]. Therefore, close follow-up of the renal function in patients with liver cirrhosis is mandatory and markers of early renal impairment are priceless in these patients [2].

Using the current markers and equations of the renal function in cirrhotic patients can be challenging. Serum creatinine, the most widely used marker, may underestimate renal impairment in patients with liver cirrhosis. Decreased hepatic production of creatine, reduced muscle mass, and malnutrition account for an increased gap between serum creatinine levels and the actual renal function [3]. High serum bilirubin levels may also interfere with the analytical methods of serum creatinine measurement, although this is no more a problem after using the modern Jaffe method autoanalyzers [4]. Inulin clearance, the standard method for measuring the glomerular filtration rate (GFR), is costly and impractical as it requires 24-hour urinary catheterization [5]. Isotopic renal scans are not less costly; they cannot be used for repeated measurements that are needed in such patients. Creatinine clearance tends to overestimate the GFR and requires accurate urine volume measurement [6]. Based on serum creatinine, Cockcroft-Gault formula and modification of diet in renal disease (MDRD) equations are of limited value in cirrhotic patients; they overestimate the GFR as well [7].

Serum cystatin C (CysC) has been proposed as a novel biomarker of the renal function [8]. Several studies have reported its value in different sets of patients [9–16]. However, only few studies have evaluated the role of serum CysC in patients with liver cirrhosis [17–19] and none of these studies have studied its role in detecting early renal impairment in these patients. In our study, we aimed at evaluating the role of serum CysC as a marker of the renal function in patients with liver cirrhosis and, more important, as an indicator of renal impairment at its early stages in such patients. We compared different markers and estimating equations, including serum CysC and CysC-based equations, to the isotopic GFR being used as the gold standard of the actual renal performance.

## 2. Methodology

### 2.1. Patients

This prospective observational study was conducted on seventy consecutive patients with liver cirrhosis who presented to the Liver Transplantation Unit, National Liver Institute, Menoufia University, as potential candidates for liver transplantation. The diagnosis of cirrhosis was based on a combination of clinical, laboratory, and ultrasonographic findings [20]. Patients aged 18–80 years were eligible for the study if they had serum creatinine less than 1.5 mg/dL. Patients were excluded if they had dehydration, sepsis, or gastrointestinal bleeding during the month before enrollment. The local ethics committee approved the study. A written informed consent was obtained from all participating patients prior to inclusion in the study.

### 2.2. Baseline Assessment

Included patients were subjected to history taking, clinical examination, abdominal ultrasonography, and baseline laboratory investigations including liver and renal tests, urine analysis, and complete blood count. Child-Pugh [21, 22] and MELD [23] scores were calculated for all patients.

### 2.3. Assessment of the Renal Function

Enrolled patients underwent ^99 m^Tc-DTPA renal scan to measure the isotopic GFR. Serum and urine samples were collected on the same day of the renal scan. Serum samples were used to measure serum creatinine, CysC, and blood urea nitrogen (BUN). The Quantikine Human Cystatin C Immunoassay (R&D Systems Inc., Minneapolis, MN, USA), an enzyme-linked immunosorbent assay (ELISA), was used to measure serum CysC on a CX7 analyzer (Beckman, Brea, CA, USA). Creatinine clearance (CCr) was calculated through the following equation: CCr = (urinary creatinine × 24 hours of urine volume)/(serum creatinine × 1440) [24]. GFR was furthermore calculated using six estimating formulae, Cockcroft-Gault formula [25], the modification of diet in renal disease (MDRD) equations using 4 and 6 variables [26, 27], and Chronic Kidney Disease Epidemiology Collaboration (CKD-EPI) equations using creatinine alone (CKD-EPI Cr) [28], CysC alone (CKD-EPI CysC), or both CKD-EPI (Cr-CysC) [29] (Table 1). Isotopic GFR was used as the reference to which all other measures and estimating formulae of the renal function were compared. In this study, the upper limit of normal for serum creatinine was 1.2 mg/dL and early renal impairment was defined as a GFR of 60–89 mL/min.

### 2.4. Statistical Analysis

Data were analyzed using IBM SPSS Statistics for Macintosh, Version 22.0 (IBM Corp., Armonk, NY, USA). Two-tailed *p* values were considered statistically significant if they were less than 0.05.

Bivariate correlations were tested using Pearson's (*r*) and Spearman's (rho) correlation coefficients for parametric and nonparametric measure of statistical dependence, respectively. The coefficient of determination (*R* ^2^) was also calculated to measure the percent of variation in one variable which could be predicted by the variation in the other variable.* R* ^2^ closer to 1 indicates a better model fit.

The ability of the studied tests and formulae to discriminate early renal impairment was evaluated using the area under the receiver operating characteristic (ROC) curve (AUC). A cutoff was then proposed to each test or formula with the most acceptable sensitivity and specificity. The following tests of diagnostic accuracy were calculated for the measures and estimates of the renal function with a 95% confidence interval: sensitivity, specificity, positive predictive value (PPV), and negative predictive value (NPV).

Bland-Altman plot was used to analyze the agreement between isotopic GFR and the other mathematical formulae of GFR. One sample* t*-test was used to test for significance of the difference between each of these formulae and isotopic GFR.

## 3. Results

The baseline characteristics and demographic and clinical data of the enrolled patients are presented in Tables 2 and 3. The study sample had predominance of males (87.1%). Etiology of the liver disease was most commonly chronic hepatitis C infection (72.9%). About 29% of the patients had hepatocellular carcinoma (HCC). Most patients were Child-Pugh class C (74.3%) and had moderate ascites (41.4%). About 64% of the patients had early renal impairment. None of the patients had proteinuria or other urine abnormalities.

**Figure 1:**
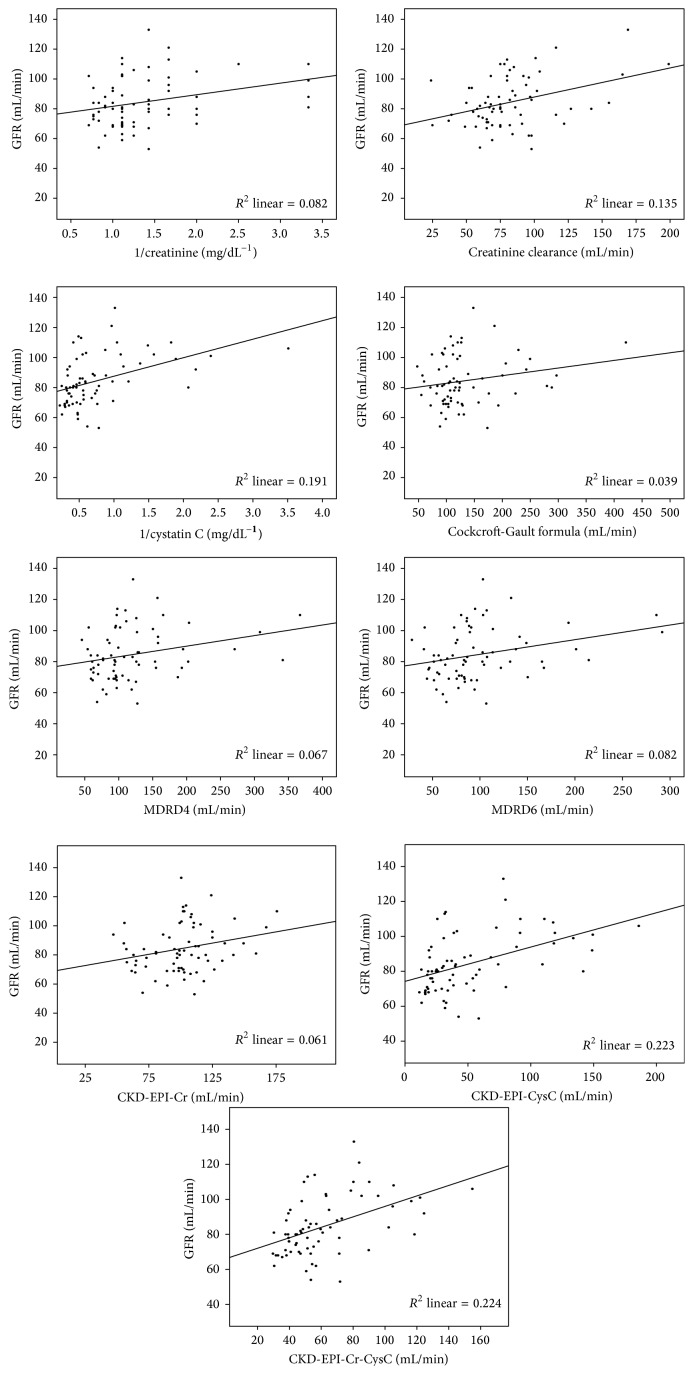
Scatter plots of the isotopic glomerular filtration rate (GFR) versus 1/creatinine, creatinine clearance, 1/cystatin C, Cockcroft-Gault formula, MDRD equations with 4 and 6 variables, CKD-EPI-Cr, CKD-EPI-CysC, and CKD-EPI-Cr-CysC.

Different measures and estimates of the renal function were correlated with the isotopic GFR using Pearson's correlation (Table 4). Serum CysC and CKD-EPI equations using CysC alone or both creatinine and CysC had the highest and most significant correlation coefficient (*r* = 0.437, 0.473, and 0.474, resp., *p* < 0.0001) (Figure 1). CKD-EPI (Cr-CysC) had the highest* R* ^2^ among all other measures.

**Figure 2:**
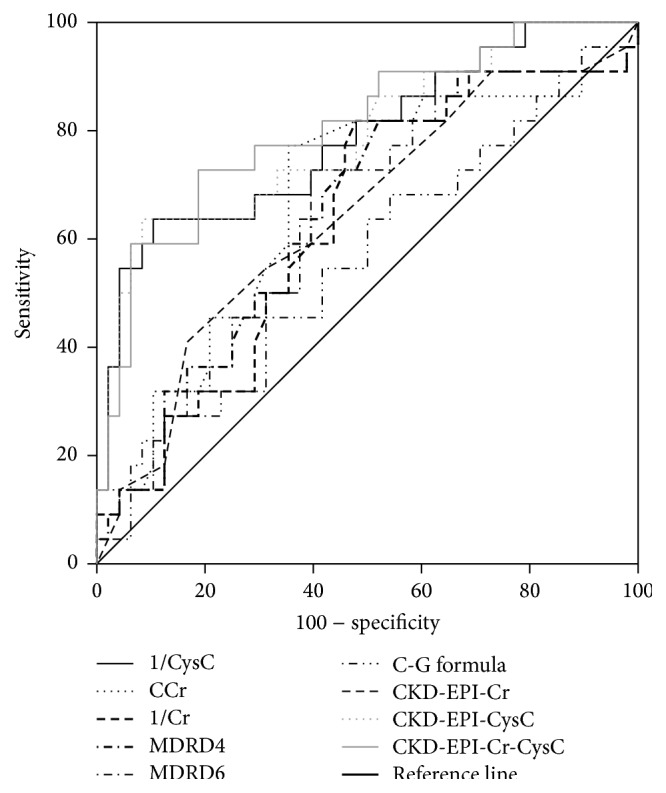
ROC curves of 1/creatinine, creatinine clearance, 1/cystatin C, MDRD equations with 4 and 6 variables, Cockcroft-Gault formula, CKD-EPI-Cr, CKD-EPI-CysC, and CKD-EPI-Cr-CysC for detecting early renal impairment.

The discriminating ability of the studied measures and estimates of the renal function in detecting early renal impairment was assessed by plotting the ROC curves (Figure 2).  Table 5 presents the AUC for each of the measures and formulae. CKD-EPI (Cr-CysC) had the largest AUC (0.808, *p* < 0.0001). We then used the ROC curves' coordinates to define cutoff values with acceptable sensitivity and specificity for the studied tests and formulae.  Table 6 shows the sensitivity, specificity, PPV, and NPV for the different measures and formulae in detecting early renal impairment according to these cutoff values. At a cutoff value of 1.2 mg/L, CysC was 89.6% sensitive and 63.6% specific in detecting early renal impairment with PPV of 84.3% and NPV of 73.7%.

**Figure 3:**
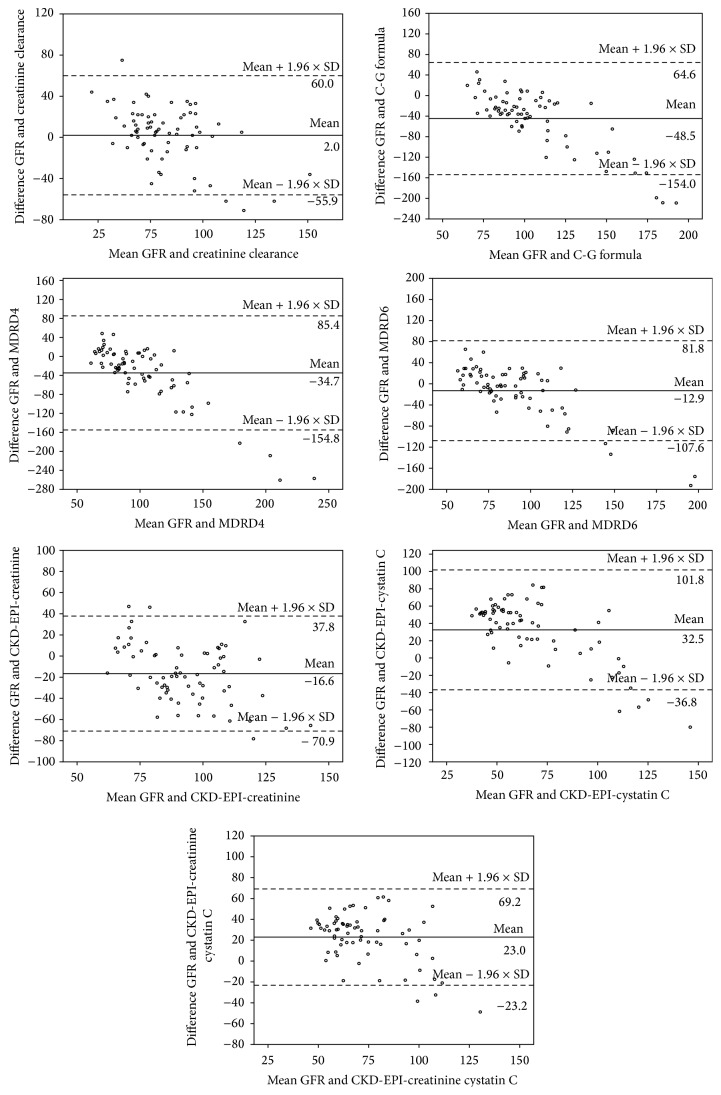
Bland-Altman plot for the agreement between isotopic GFR and the other GFR estimating formulae.

The Bland-Altman plot was used to describe agreement between isotopic GFR and different mathematical formulae (Table 7 and Figure 3). The mean difference with CCr was 2.0429, which was not statistically significant (*p* = 0.565).

## 4. Discussion

This study showed that serum CysC and CysC-based equations were the best measures that correlated with the isotopic GFR, compared to other measures and equations that have been evaluated in the study. This confirms the results of the previous studies that came up with similar conclusions [18, 30–32]. Several studies have reported the superior diagnostic accuracy of serum CysC and CysC-based formulae over other markers and equations in detecting moderate and severe renal impairment in patients with liver cirrhosis [18, 33, 34]. However, since patients with liver cirrhosis are extremely sensitive to the modest decreases in the GFR that can markedly impact their survival [35], it is of great clinical importance to identify markers that can detect renal impairment at its early stage in these patients. Using Pearson's correlation, our study found that CKD-EPI (Cr-CysC) formula was the most accurate marker, with the largest AUC, in detecting early renal impairment in patients with liver cirrhosis. This finding is novel, putting CysC and CysC-based equations on the top of the list of markers and estimating formulae of the renal function in such patients. The results also showed that serum CysC was the most sensitive (89.6%) measure in detecting early renal impairment at a cutoff value of 1.2 mg/L with acceptable specificity (63.6%).

One strong limitation of serum creatinine and creatinine-based equations is that serum creatinine lags behind a decreasing GFR [36]. So they are not accurately reflecting the present status of the renal function of the patient, a limitation that has been overcome by serum CysC that proved to accurately reflect the early stages of renal impairment according to the results of this study.

However, Bland-Altman plot analysis showed disputing results. Estimated CCr was the only measurement that had significant agreement with isotopic GFR. This contradiction may be explained as a correlation coefficient between two measures might be highly correlated; yet, there could be substantial differences in the two measurements across their range of values. Also, results of Bland-Altman method might be disturbed when there is heterogeneous bias with significant heteroscedastic error [37].

The results of this study are robust even though the sample size was relatively small because we have used the isotopic GFR as the reference to which all other measures and formulae were compared. Therefore, in contrast to some previous studies that used CCr as reference [33, 38], the results of our study can be generalized over a larger scale. Still, we recommend further studies to be designed to confirm and validate the results of this study. Future studies should formulate a general predictive model of the renal function in patients with liver cirrhosis based on serum CysC levels, a model that can make the isotopic GFR dispensable.

In conclusion, finding a good marker of the renal function is crucial to the survival of patients with liver cirrhosis, given the huge impact of the renal impairment on the prognosis of these patients. Our study found that serum CysC and CysC-based formulae were not only the best measures that reflected the actual renal performance in cirrhotic patients, but also the most accurate ones in detecting early stages of renal impairment in these patients.

## Figures and Tables

**Table 1:** GFR-estimating equations used in the study.

Cockcroft-Gault formula	140 – age (years) × weight (kg)/(Scr × 72) × 0.85 if female
MDRD equations using 4 variables	186 × Scr^−1.154^ × age (years)^−0.203^ × 0.742 if female × 1.212 if black

MDRD equations using 6 variables	170 × Scr^−0.999^ × age (years)^−0.176^ × BUN (mg/dL)^−0.170^ × albumin (g/dL)^0.318^ × 0.762 if female × 1.180 if black

The CKD-EPI creatinine equation	141 × min(Scr/*κ*, 1)^*α*^ × max(Scr/*κ*, 1)^−1.209^ × 0.993^Age^ [×1.018 if female] [×1.159 if black], where *α* is −0.329 for females and −0.411 for males

The CKD-EPI cystatin C equation	133 × min(Scys/0.8, 1)^−0.499^ × max(Scys/0.8, 1)^−1.328^ × 0.996^Age^ [×0.932 if female]

The CKD-EPI creatinine–cystatin C equation	135 × min(Scr/*κ*, 1)^*α*^ × max(Scr/*κ*, 1)^−0.601^ × min(Scys/0.8, 1)^−0.375^ × max(Scys/0.8, 1)^−0.711^ × 0.995^Age^ [×0.969 if female] [×1.08 if black], where *α* is −0.248 for females and −0.207 for males

*κ* is 0.7 for females and 0.9 for males, max is the maximum of Scr/*κ* or 1, min is the minimum of Scr/*κ* or 1, Scr is serum creatinine in (mg/dL), and Scys is serum cystatin C in (mg/L).

**Table 2:** Baseline characteristics of the enrolled patients.

	Mean ± SD	Range
Age (years)	47.4 ± 9.3	18–75
Weight (kg)	78.2 ± 14.7	48–110
Total bilirubin (mg/dL)	3.9 ± 3.2	0.6–17.5
Albumin (g/dL)	2.6 ± 0.5	1.7–3.5
Isotopic GFR (mL/min)	84.5 ± 16.6	53–133
BUN (mg/dL)	19.3 ± 10.8	4.7–72.3
Creatinine (mg/dL)	0.8 ± 0.3	0.3–1.4
Creatinine clearance (mL/min)	82.4 ± 31.3	24–199
Cystatin C (mg/L)	1.9 ± 1	0.3–4.5
Child-Pugh score	10.7 ± 1.7	7–14
MELD score	16.2 ± 4.9	8–31
MDRD 4 (mL/min)	119.2 ± 63.5	45.7–367
MDRD 6 (mL/min)	97.4 ± 50.4	28.4–291.7
Cockcroft-Gault formula (mL/min)	132.9 ± 65	47.9–421.1
CKD-EPI-Cr (mL/min)	101.0 ± 26.7	47.1–175.6
CKD-EPI-CysC (mL/min)	52 ± 40	11.4–185.9
CKD-EPI-Cr-CysC (mL/min)	61.5 ± 26.3	29.7–154.8

BUN, blood urea nitrogen; CKD-EPI, Chronic Kidney Disease Epidemiology Collaboration; GFR, glomerular filtration rate; MDRD, modification of diet in renal disease equation with 4 and 6 variables; MELD, model for end-stage liver disease; SD, standard deviation.

**Table 3:** Demographic and clinical data of the enrolled patients.

	*n* (%)
Sex	
Males	61 (87.1)
Females	9 (12.9)
Etiology of liver disease	
Chronic hepatitis C	51 (72.9)
Chronic hepatitis B	12 (17.1)
Budd-Chiari syndrome	2 (2.9)
Unknown etiology	5 (7.1)
HCC	
No	50 (71.4)
Yes	20 (28.6)
Ascites	
No	9 (12.9)
Mild	23 (32.8)
Moderate	29 (41.4)
Marked	9 (12.9)
Child-Pugh class	
B	18 (25.7)
C	52 (74.3)
Renal impairment	
No (GFR ≥90 mL/min)	22 (31.4)
Early (GFR 60–89 mL/min)	45 (64.3)
Advanced (GFR 30–59 mL/min)	3 (4.3)

GFR, glomerular filtration rate; HCC, hepatocellular carcinoma.

**Table 4:** Pearson's correlation of the variables with the isotopic GFR.

	*r*	*p*
1/creatinine (mg/dL^−1^)	0.287	0.016
Creatinine clearance (mL/min)	0.367	0.002
1/cystatin C (mg/L^−1^)	0.437	<0.0001
MDRD 4	0.260	0.030
MDRD 6	0.286	0.017
Cockcroft-Gault formula	0.198	0.100
CKD-EPI-Cr	0.247	0.039
CKD-EPI-CysC	0.473	<0.0001
CKD-EPI-Cr-CysC	0.474	<0.0001

CKD-EPI, Chronic Kidney Disease Epidemiology Collaboration; Cr, creatinine; CysC, cystatin C; MDRD, modification of diet in renal disease equation with 4 and 6 variables; *r*, Pearson's correlation coefficient.

**Table 5:** Area under the curve for detecting early renal impairment for studied tests and formulae.

Variable	AUC	95% CI	*p*
1/Cr	0.642	0.500–0.784	0.058
CCr	0.674	0.533–0.815	0.020
1/CysC	0.785	0.663–0.907	<0.0001
MDRD 4	0.646	0.506–0.787	0.051
MDRD 6	0.644	0.503–0.784	0.054
C-G formula	0.562	0.413–0.710	0.411
CKD-EPI-Cr	0.632	0.491–0.773	0.078
CKD-EPI-CysC	0.788	0.667–0.909	<0.0001
CKD-EPI-Cr-CysC	0.808	0.695–0.921	<0.0001

AUC, area under the curve; C-G formula, Cockcroft-Gault formula; CKD-EPI, Chronic Kidney Disease Epidemiology Collaboration; CI, confidence interval; Cr, creatinine; CCr, creatinine clearance; CysC, cystatin C; MDRD, modification of diet in renal disease equation with 4 and 6 variables.

**Table 6:** Cutoffs of measures and estimates for detection of early renal impairment with their measures of diagnostic accuracy.

	Cutoff of early RI	Sensitivity (%)	Specificity (%)	PPV (%)	NPV (%)
Cr	0.75 mg/dL	68.8	54.5	76.7	44.4
CCr	77 mL/min	77.3	64.6	54.5	60.9
CysC	1.2 mg/L	89.6	63.6	84.3	73.7
MDRD 4	96.8 mL/min	72.7	54.2	57.3	62.7
MDRD 6	85.5 mL/min	72.7	60.4	54.6	59.2
C-G formula	119.3 mL/min	54.5	58.3	48.3	47.8
CKD-EPI-Cr	99.8 mL/min	77.3	55.2	63.3	70.9
CKD-EPI-CysC	38.7 mL/min	72.7	66.7	68.6	71.0
CKD-EPI-Cr-CysC	52.8 mL/min	77.3	61.4	66.7	73.0

C-G formula, Cockcroft-Gault formula; CKD-EPI, Chronic Kidney Disease Epidemiology Collaboration; Cr, creatinine; CCr, creatinine clearance; CysC, cystatin C; MDRD, modification of diet in renal disease equation with 4 and 6 variables; NPV, negative predictive value; PPV, positive predictive value; RI, renal impairment.

**Table 7:** Bland-Altman plot analysis for the agreement between isotopic GFR and the other GFR estimating formulae.

	Mean ± SD	*p*	95% CI
CCr	2.04 ± 29.6	0.565	−5.0–9.1
C-G formula	−44.7 ± 55.8	<0.0001	−58.1–−31.3
MDRD 4	−34.7 ± 61.3	<0.0001	−49.3–−20.1
MDRD 6	−12.9 ± 48.3	0.028	−24.5–−1.4
CKD-EPI-Cr	−16.6 ± 27.7	<0.0001	−23.2–−9.9
CKD-EPI-CysC	32.5 ± 35.3	<0.0001	24.1–40.9
CKD-EPI-Cr-CysC	23 ± 23.6	<0.0001	17.4–28.6

C-G formula, Cockcroft-Gault formula; CKD-EPI, Chronic Kidney Disease Epidemiology Collaboration; Cr, creatinine; CCr, creatinine clearance; CysC, cystatin C; MDRD, modification of diet in renal disease equation with 4 and 6 variables.
